# COVID-19 and subsequent risk of coded diabetic retinopathy in patients with type 2 diabetes: a multicenter propensity score-matched cohort study

**DOI:** 10.3389/fendo.2026.1914361

**Published:** 2026-09-11

**Authors:** Kuo-Chuan Hung, Ting-Sian Yu, Su-Zhen Wu, I-Wen Chen

**Affiliations:** 1 Department of Anesthesiology, Chi Mei Medical Center, Tainan, Taiwan; 2 Department of Anesthesiology, E-Da Hospital, I-Shou University, Kaohsiung, Taiwan; 3 Department of Anesthesiology, Chi Mei Hospital, Liouying, Tainan, Taiwan

**Keywords:** COVID-19, diabetic macular edema, diabetic retinopathy, landmark analysis, propensity score matching, real-world data, SARS-CoV-2, type 2 diabetes mellitus

## Abstract

**Background:**

Evidence remains limited regarding whether coronavirus disease 2019 (COVID-19) is associated with the subsequent risk of EHR-recorded diabetic retinopathy (DR) during available follow-up in patients with type 2 diabetes mellitus (T2DM). We evaluated the risk of EHR-recorded DR and clinically relevant DR subtypes after severe acute respiratory syndrome coronavirus 2 (SARS-CoV-2) infection during follow-up within a maximum 5-year analytic window.

**Methods:**

This retrospective multicenter cohort study used the TriNetX Global Collaborative Network. Adults with T2DM were classified into COVID-19 and control groups between January 1, 2020, and June 30, 2022. A 6-month landmark design was applied to reduce early outcome misclassification. After 1:1 propensity score matching, 773,768 patients were included in each group. The primary outcome was electronic health record (EHR)-recorded incident DR. Secondary outcomes included EHR-recorded DR subtypes (non-proliferative DR, proliferative DR, and diabetic macular edema) and glycemic events, including hyperglycemia (glucose ≥180 mg/dL), poor glycemic control (HbA1c ≥9%), and hypoglycemia (glucose <70 mg/dL). Secondary and supportive analyses were exploratory and were not adjusted for multiplicity.

**Results:**

Median follow-up was 1,487 days in the COVID-19 group and 1,634 days in controls. EHR-recorded incident DR occurred more frequently in the COVID-19 group than in the control group (2.17% vs. 1.64%; absolute risk difference, 0.53 percentage points; hazard ratio [HR], 1.51; 95% confidence interval [CI], 1.48–1.55; p<0.001). COVID-19 was also associated with higher risks of non-proliferative DR (HR, 1.53; 95% CI, 1.49–1.58), proliferative DR (HR, 1.67; 95% CI, 1.57–1.78), and diabetic macular edema (HR, 1.75; 95% CI, 1.67–1.83). In addition, COVID-19 was associated with increased risks of glycemic events. The associations remained consistent across sensitivity analyses, including survivor-only, healthcare-visit-restricted, eye-care-visit-restricted, 1-year landmark, and 2-year landmark models. Exploratory severe-COVID analysis showed a similar direction of association.

**Conclusions:**

Among adults with T2DM, COVID-19 was associated with an increased risk of EHR-recorded incident DR, including coded vision-threatening subtypes (proliferative DR and diabetic macular edema), during follow-up. These findings suggest a possible association between COVID-19 and subsequent EHR-recorded DR during available follow-up, although residual confounding and differential ophthalmic surveillance cannot be excluded.

## 1 Introduction

Diabetic retinopathy (DR) is a common microvascular complication of type 2 diabetes mellitus (T2DM) and a major cause of preventable vision loss (1–4). Because DR reflects progressive retinal microvascular injury from long-term metabolic dysfunction and may remain asymptomatic even when vision-threatening disease is present, timely risk stratification and sustained ophthalmic surveillance are essential to enable early referral and treatment before irreversible visual impairment occurs (5, 6). Coronavirus disease 2019 (COVID-19) is now recognized as a systemic disease that may lead to persistent vascular, inflammatory, thrombotic, and metabolic abnormalities beyond the initial infection (7–10). With the transition from the acute phase of the pandemic to the post-pandemic era, increasing attention has focused on the long-term health consequences of severe acute respiratory syndrome coronavirus 2 (SARS-CoV-2) infection. Prior studies have reported elevated risks of new-onset diabetes and post-acute glycemic dysregulation after SARS-CoV-2 infection (10–13), and emerging evidence suggests that some individuals continue to experience worsening glycemic control, including higher HbA1c levels and increased requirements for glucose-lowering therapy, months after recovery (14, 15). Because chronic hyperglycemia is a key driver of diabetic microvascular complications, these observations raise the hypothesis that SARS-CoV-2 infection may be associated with downstream diabetes-related outcomes, including DR. However, whether COVID-19-related dysglycemia translates into DR development or progression remains uncertain.

However, evidence regarding COVID-19 and incident DR remains limited. Existing studies have primarily examined the indirect effects of the pandemic on DR care, including disruptions in screening, reduced access to ophthalmic services, and delays in treatment (16–18), rather than the development of new-onset DR following SARS-CoV-2 infection. These pandemic-related changes in DR screening and ophthalmic care also create an important methodological challenge, because differential eye-care access and diagnostic opportunity may influence ascertainment of coded DR outcomes. The main available cohort evidence is largely limited to single–health-system data with follow-up of up to approximately three years and limited subtype-specific assessment (19), leaving uncertainty regarding longer-term and clinically relevant DR outcomes such as proliferative DR and diabetic macular edema. Therefore, it remains unclear whether the excess risk, if present, is confined to the early post-infection period or persists over longer follow-up. It is also uncertain whether COVID-19 is associated with more advanced retinal outcomes, such as proliferative DR or diabetic macular edema, which are more directly linked to vision-threatening disease (20).

Clarifying this question is clinically relevant in the post-pandemic era, given the large number of patients with T2DM who have recovered from SARS-CoV-2 infection. Identifying whether COVID-19 is associated with subsequent EHR-recorded DR during available follow-up may help clarify its potential relevance to retinal risk assessment in this population. We therefore conducted a multicenter, propensity score–matched cohort study using the TriNetX Global Collaborative Network to evaluate the risk of incident DR and its subtypes within an up to 5-year analytic window among adults with T2DM following SARS-CoV-2 infection.

## 2 Methods

### 2.1 Study design and data source

This retrospective multicenter cohort study used the TriNetX Global Collaborative Network, a federated research platform that provides access to de-identified electronic health records from participating healthcare organizations. At the time of data extraction, the query included data from 177 participating healthcare organizations. Organization-level geographic distribution was not available in the aggregate analytic output provided to investigators. The database contains longitudinal information on demographics, diagnoses, procedures, medications, laboratory results, and healthcare encounters. The study was designed to evaluate whether SARS-CoV-2 infection was associated with the subsequent risk of EHR-recorded DR during available follow-up among adults with type 2 diabetes mellitus (T2DM). Because all analyses were performed using de-identified and aggregated data within the TriNetX platform, individual-level patient identifiers were not available to investigators. The study was approved by the Institutional Review Board of Chi Mei Medical Center, and the requirement for informed consent was waived. All procedures were conducted in accordance with the Declaration of Helsinki.

### 2.2 Study population and exposure definition

Eligible patients were adults aged 18 years or older with T2DM. To ensure baseline healthcare-system engagement and reduce differential capture of comorbidities and outcomes, all patients in both cohorts were required to have at least one recorded healthcare visit within the 5 years before the index date. The exposure cohort included patients with T2DM who had evidence of SARS-CoV-2 infection during the primary exposure period from January 1, 2020, to June 30, 2022. SARS-CoV-2 infection was defined by either an ICD-10-CM diagnosis code for COVID-19 or a positive SARS-CoV-2 RNA laboratory record. The control cohort included patients with T2DM during the same calendar period who had no recorded COVID-19 diagnosis and no positive SARS-CoV-2 RNA test in the available electronic health record.

The index date was defined as the first date on which a patient met the cohort-defining criteria during the study period. For the COVID-19 group, this corresponded to the first qualifying COVID-19 diagnosis or positive SARS-CoV-2 RNA test in a patient with T2DM. For the control group, the index date was derived from the qualifying T2DM record during the same calendar period in patients without recorded COVID-19. When multiple qualifying T2DM records were available for a control patient, the first record that satisfied the cohort-entry criteria during the eligibility period was assigned as the index date. Exact calendar-month or calendar-quarter matching of index dates was not performed because this function was not available within the aggregate TriNetX analytic workflow used for this study. However, both cohorts were required to enter the study during the same eligibility period from January 1, 2020, to June 30, 2022, which helped reduce major calendar-period differences between groups. Controls were classified according to baseline exposure status and were retained in the control cohort if COVID-19 was recorded later during follow-up.

To reduce prevalent-outcome contamination and reduce the likelihood of pre-existing coded retinal disease at baseline, patients with pre-existing T2DM with ophthalmic complications were excluded if recorded before the index date. Additional exclusions were applied to reduce heterogeneity in diabetes phenotype and major systemic illness. These included type 1 diabetes mellitus, gestational diabetes mellitus, stage 4–5 chronic kidney disease, end-stage renal disease, dependence on renal dialysis, and bariatric surgery status. Patients with prior retinal disease or retinal procedures were also excluded, including blindness or low vision, retinal vascular occlusion, retinal detachment or breaks, other retinal disorders, intravitreal pharmacologic injection, vitrectomy, retinal or choroidal photocoagulation, and procedures for extensive or progressive retinopathy.

### 2.3 Landmark analysis

A 6-month landmark design was used to reduce the possibility that DR diagnoses recorded soon after COVID-19 represented pre-existing but previously undetected disease or short-term ascertainment related to acute infection. Patients who developed DR or died during the first 6 months after the index date were excluded to minimize reverse causation and immortal time bias and to ensure that only patients at risk for incident DR entered the analytic cohort. Follow-up for the primary analysis began after completion of the 6-month landmark period and continued through a maximum 5-year analytic window. Patients were followed until the first occurrence of the outcome of interest, death when applicable, loss to follow-up as defined by the last available record in the TriNetX database, or the end of the 5-year follow-up period, whichever occurred first. The diagnostic codes are summarized in **Supplementary Table 1**.

### 2.4 Propensity score matching

To reduce baseline confounding, we performed 1:1 propensity score matching between the COVID-19 and control groups using a greedy nearest-neighbor algorithm with a caliper of 0.1 pooled standard deviations of the propensity score logit. Covariates were selected *a priori* based on clinical relevance to COVID-19 risk, diabetes severity, healthcare utilization, and DR risk. All baseline covariates used for propensity score matching were assessed during the 5-year pre-index lookback period and were required to be recorded before the index date. These included demographics, race/ethnicity, cardiometabolic and systemic comorbidities, diabetes-related complications, ocular comorbidities, medication use, vaccination status, and laboratory markers of glycemic control, renal function, anemia, nutritional status, and hypoglycemia. For diagnoses, procedures, and medications, absence of a record was interpreted as absence of a recorded condition, procedure, or medication use within the pre-index lookback window. Laboratory covariates were encoded as recorded threshold-defined features within the TriNetX platform, and missing laboratory values were not imputed. Therefore, absence of a recorded threshold-defined laboratory value should not be interpreted as confirmed normal physiology. The availability and missingness of laboratory covariates are summarized in **Supplementary Table 2**. Covariate balance was assessed using standardized mean differences (SMDs), with SMD <0.10 indicating acceptable balance, and was further visually evaluated using propensity score density plot. Variables included in the propensity score model are listed in **Supplementary Table 3**.

### 2.5 Outcomes

The primary outcome was EHR-recorded incident DR, identified using ICD-10-CM codes for T2DM with DR, including unspecified DR, non-proliferative DR, and proliferative DR. Secondary outcomes included EHR-recorded DR subtypes (non-proliferative DR, proliferative DR, and diabetic macular edema), hyperglycemia (serum, plasma, or blood glucose ≥180 mg/dL), poor glycemic control (hemoglobin A1c [HbA1c] ≥9%), and hypoglycemia (glucose <70 mg/dL). These outcomes were used to contextualize whether post-COVID glycemic instability might parallel the observed retinal risk pattern. Additional supportive outcomes included all-cause mortality, age-related cataract, and any healthcare visit.

The retinal outcomes were based on coded clinical diagnoses rather than standardized retinal imaging-confirmed or adjudicated incident DR. ICD-10-CM code sets were selected at the parent-concept level in the TriNetX hierarchy and included all available descendant codes, including laterality-specific, severity-specific, and eye-unspecified extensions when present. Non-proliferative DR, proliferative DR, and diabetic macular edema were analyzed as separate, non-mutually exclusive outcomes; therefore, diabetic macular edema could overlap with non-proliferative or proliferative DR. Unspecified DR codes were included in the overall DR endpoint but were not analyzed as a separate subtype.

Cataract was evaluated as a negative-control ocular outcome to assess outcome-specific detection bias. Because cataract is a common age-related eye disease that is not expected to be specifically associated with COVID-19–related retinal microvascular injury, an increased cataract incidence in the COVID-19 group would suggest that more frequent ophthalmic examinations or greater opportunities for eye disease detection, rather than a true biological effect, might partly explain the observed association with DR. In contrast, healthcare visits were assessed as a broader marker of overall healthcare utilization and surveillance intensity, reflecting differences in patients’ contact with the healthcare system irrespective of ophthalmic care. Together, these measures helped distinguish outcome-specific detection bias from general surveillance bias. The definitions of all outcomes are provided in **Supplementary Table 4**.

### 2.6 Sensitivity, subgroup analyses, multivariable Cox regression

Sensitivity analyses were performed to evaluate the robustness of the association between COVID-19 and retinal outcomes. Model I included only patients who survived during follow-up to assess whether differential mortality influenced the observed association. Model II included only patients who had at least one healthcare visit during follow-up to reduce the potential influence of differential healthcare utilization and outcome ascertainment. Model III used a 1-year landmark design, and Model IV used a 2-year landmark design, to evaluate whether the association persisted after excluding early post-index events. These landmark analyses were interpreted as sensitivity analyses and were not considered sufficient to eliminate residual ascertainment or selection bias. To further address potential differential ophthalmic surveillance and DR ascertainment, we additionally performed a sensitivity analysis restricted to patients with at least one eye-care visit during follow-up. Eye-care visits were defined using the TriNetX ophthalmology services and procedures hierarchy term 1012793, including available descendant ophthalmology service/procedure concepts captured within the platform. Because this is a broad hierarchy-based measure, it should be interpreted as a proxy for ophthalmic contact rather than a direct measure of retinal imaging intensity, optical coherence tomography, fundus photography, or standardized diabetic retinopathy screening.

Prespecified subgroup analyses were conducted for the primary outcome according to sex, age group (18–65 vs. >65 years), chronic kidney disease, hypertension, HbA1c ≥9%, hyperlipidemia, obesity, and smoking history. Multivariable Cox proportional hazards regression was performed independently of propensity score matching to evaluate the association between COVID-19 and incident DR. The model adjusted for major baseline demographic and clinical covariates, including age, sex, race, diabetes-related complications, cardiovascular and systemic comorbidities, and lifestyle-related factors. This analysis was intended as a supportive robustness analysis of the COVID-19 association independent of propensity score matching, rather than as an etiologic risk-factor model for individual covariates. Therefore, individual covariate coefficients were interpreted as mutually adjusted conditional associations and not as independent causal, protective, or risk-factor estimates, because they may be influenced by coding patterns, multicollinearity, overadjustment, collider bias, and competing risks.

### 2.7 Severe-COVID and late-period analyses

To explore whether patients with more clinically severe COVID-19 showed a similar retinal risk pattern, we performed a supportive severe-COVID analysis comparing patients with severe COVID-19 with matched controls. Severe COVID-19 was defined as a COVID-19 index event with a concurrent hospitalization or intensive care unit admission code recorded on the index date. Hospitalization and intensive care unit admission were not analyzed as separate severity strata; the presence of either code was used to classify patients as having severe COVID-19. This analysis was interpreted as supportive rather than as a formal exposure-gradient or dose–response analysis. Detailed inpatient variables, including corticosteroid exposure, mechanical ventilation, acute kidney injury, and inpatient hyperglycemia, were not available for this analysis within the aggregate TriNetX workflow. Baseline ophthalmology services and procedures were included in the propensity score model for the severe-COVID comparison to account for pre-index ophthalmic surveillance.

A supportive late-period analysis was also performed to address potential differences by pandemic period and circulating viral variants. This analysis was restricted to patients with first SARS-CoV-2 infection between July 2022 and June 2023. Patients in the late COVID-19 group were compared with matched controls who had no history of COVID-19. Follow-up was based on the available observation time after cohort entry in this later calendar period. The same retinal, metabolic, ocular comparator, healthcare-utilization, and mortality outcomes were assessed.

### 2.8 Descriptive competing-risk analysis

Because death may preclude subsequent diagnosis or recording of DR, we performed an additional descriptive competing risk analysis to contextualize the primary findings. This analysis was conducted in the unmatched cohort and treated all-cause mortality as a competing event, with DR as the event of interest. The cumulative incidence was estimated using the Aalen–Johansen method and reported as the cumulative incidence at the end of the follow-up window. This analysis was considered supplemental and descriptive because the TriNetX platform provided competing-risk functionality only for the unmatched aggregate cohort workflow used in this study. Matched-cohort Aalen–Johansen curves, matched number-at-risk tables, and Fine–Gray subdistribution hazard models could not be generated within the platform, and individual-level event and censoring data were not available for external competing-risk modeling.

### 2.9 Statistical methods

Because not all patients had complete 5-year observation, the estimates should be interpreted as associations observed during available follow-up within a maximum 5-year analytic window rather than as complete 5-year cumulative risk estimates for every patient. The data were extracted from the TriNetX platform on May 20, 2026. Because TriNetX provides only de-identified aggregate output from a federated electronic health record network, exact patient-level censoring dates, the date of the last qualifying patient included in each analytic cohort, and complete number-at-risk tables across all analytic cohorts were not directly retrievable by the investigators. Therefore, we reported the available follow-up summaries generated by the platform, including mean and median follow-up where available, together with the analytic cohort size for each analysis.

All primary outcome analyses were performed after propensity score matching. Event counts and percentages were summarized for each cohort. Time-to-event outcomes were analyzed using Cox proportional hazards models, and associations were reported as hazard ratios (HRs) with 95% confidence intervals (CIs). For retinal outcomes, these Cox models were interpreted as cause-specific hazard models, with death before DR treated as a censoring event. The proportional hazards assumption was assessed using the platform-generated Cox proportionality diagnostics available within TriNetX. To assess the potential influence of unmeasured confounding, E-values were calculated for the primary outcome estimates. Missing data were not imputed; analyses were conducted using available data within the TriNetX platform. Two-sided p values were reported, with p <0.05 considered statistically significant for the primary outcome. Propensity score matching was repeated separately for each sensitivity, landmark, severe-COVID, and late-period analysis using the eligible analytic cohort for that analysis, rather than simply subsetting the original matched primary cohort. Secondary, supportive, sensitivity, subgroup, severe-COVID, and late-period analyses were considered exploratory, and no adjustment for multiple comparisons was performed; therefore, these findings should be interpreted as hypothesis-generating. All analyses were performed within the TriNetX Analytics platform.

## 3 Results

### 3.1 Patient selection and baseline characteristics

**Figure 1:**
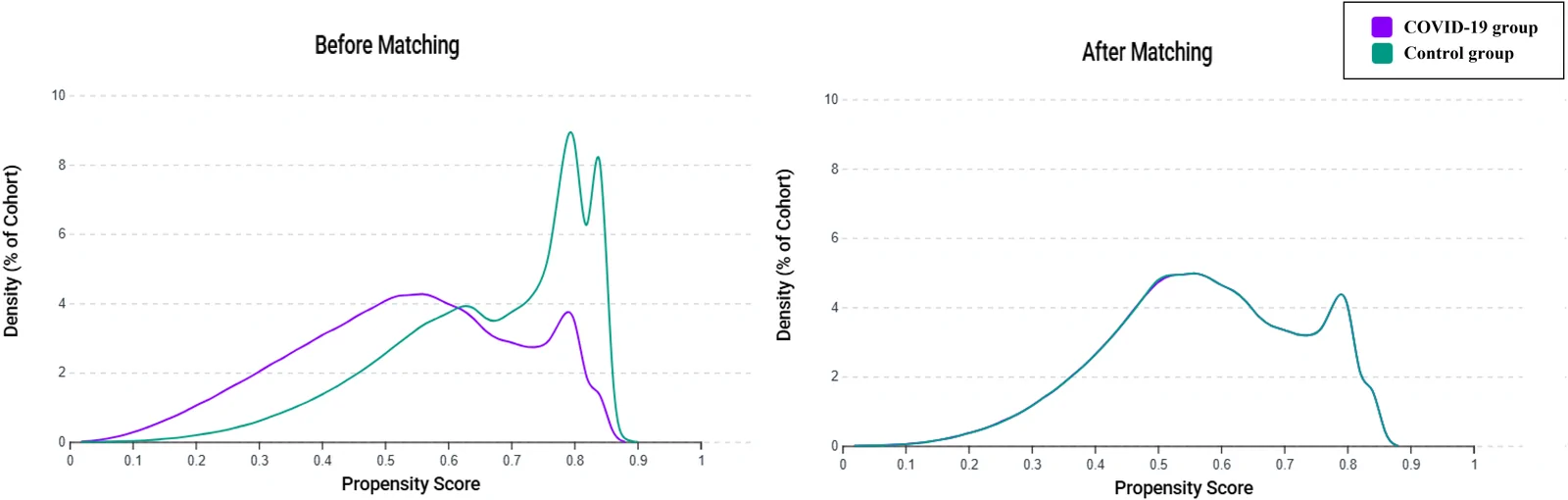
Propensity score distributions before and after matching. Propensity score density plots comparing patients with type 2 diabetes with COVID-19 and matched controls before and after 1:1 propensity score matching. Before matching, the two cohorts showed partial separation in propensity score distributions. After matching, the distributions showed substantial overlap, indicating improved covariate balance between groups.

Before propensity score matching, 902,827 patients were included in the COVID-19 group and 1,486,436 in the control group (**Table 1**). Patients with COVID-19 had higher rates of several comorbidities, diabetes-related complications, insulin use, and metabolic indicators than controls. After 1:1 propensity score matching, 773,768 patients remained in each group. Baseline characteristics were well balanced, with all standardized mean differences below 0.10. The mean age was 62.4 years in both groups, and women accounted for 49.7% and 49.1% of the COVID-19 and control groups, respectively. Major comorbidities, diabetes-related complications, medications, vaccination status, and laboratory indicators were comparable after matching, and propensity score distributions showed improved overlap between groups (**Figure 1**).

**Table 1:** Baseline characteristics of patients with type 2 diabetes with and without COVID-19 before and after propensity score matching.

Variables	Before matching			After matching		
	COVID-19 group (n = 902,827)	Control group (n = 1,486,436)	SMD	COVID-19 group (n = 773,768)	Control group (n = 773,768)	SMD
Patient characteristics						
Age at index (years)	62.6 ± 14.3	62.6 ± 13.9	0.002	62.4 ± 14.5	62.4 ± 13.9	0.001
Female	449334 (49.8)	724768 (48.8)	0.020	384326 (49.7)	379677 (49.1)	0.012
BMI>30 kg/m^2^	436741 (48.4)	482256 (32.4)	0.329	348718 (45.1)	359415 (46.5)	0.028
White	552716 (61.2)	766679 (51.6)	0.195	460979 (59.6)	472066 (61.0)	0.029
Black or African American	164966 (18.3)	220652 (14.8)	0.092	137116 (17.7)	139896 (18.1)	0.009
Asian	38889 (4.3)	82382 (5.5)	0.057	35999 (4.7)	38771 (5.0)	0.017
Comorbidities and health services						
Essential (primary) hypertension	576761 (63.9)	656882 (44.2)	0.403	461700 (59.7)	470220 (60.8)	0.022
Disorders of lipoprotein metabolism and other lipidemias	505009 (55.9)	592055 (39.8)	0.327	404467 (52.3)	410426 (53.0)	0.015
Overweight and obesity	295777 (32.8)	274479 (18.5)	0.332	221423 (28.6)	222090 (28.7)	0.002
Neoplasms	245139 (27.2)	253117 (17.0)	0.246	186126 (24.1)	186849 (24.1)	0.002
Ischemic heart diseases	204056 (22.6)	189504 (12.7)	0.260	144248 (18.6)	142779 (18.5)	0.005
Nicotine dependence	129095 (14.3)	105155 (7.1)	0.235	89396 (11.6)	88040 (11.4)	0.006
Diseases of liver	109132 (12.1)	97189 (6.5)	0.192	76599 (9.9)	74784 (9.7)	0.008
Chronic kidney disease (CKD)	108307 (12.0)	92207 (6.2)	0.202	73859 (9.5)	72932 (9.4)	0.004
Cerebrovascular diseases	97156 (10.8)	87333 (5.9)	0.178	67036 (8.7)	65977 (8.5)	0.005
T2DM with neurological complications	94848 (10.5)	78152 (5.3)	0.196	64250 (8.3)	63396 (8.2)	0.004
T2DM with kidney complications	89931 (10.0)	80906 (5.4)	0.170	62107 (8.0)	61315 (7.9)	0.004
Heart failure	104022 (11.5)	68481 (4.6)	0.256	62001 (8.0)	59304 (7.7)	0.013
Other chronic obstructive pulmonary disease	95483 (10.6)	65809 (4.4)	0.235	58148 (7.5)	55866 (7.2)	0.011
Peripheral vascular diseases	50666 (5.6)	39962 (2.7)	0.147	33004 (4.3)	32196 (4.2)	0.005
Age-related cataract	42040 (4.7)	50157 (3.4)	0.065	32955 (4.3)	33149 (4.3)	0.001
T2DM with circulatory complications	44425 (4.9)	34138 (2.3)	0.141	28868 (3.7)	28099 (3.6)	0.005
Glaucoma	27102 (3.0)	32279 (2.2)	0.052	21239 (2.7)	21459 (2.8)	0.002
Alcohol related disorders	34964 (3.9)	22639 (1.5)	0.145	21076 (2.7)	20127 (2.6)	0.008
Malnutrition	17361 (1.9)	6972 (0.5)	0.134	7536 (1.0)	6680 (0.9)	0.012
Medications						
Antilipemic agents	444065 (49.2)	490731 (33.0)	0.333	348168 (45.0)	352724 (45.6)	0.012
Biguanides	350923 (38.9)	421360 (28.3)	0.224	285933 (37.0)	293941 (38.0)	0.021
Insulins and analogues	279849 (31.0)	237212 (16.0)	0.361	199469 (25.8)	196690 (25.4)	0.008
Sulfonylureas	133643 (14.8)	162597 (10.9)	0.116	108189 (14.0)	110893 (14.3)	0.010
Dipeptidyl peptidase 4 (DPP-4) inhibitors	81434 (9.0)	109171 (7.3)	0.061	67540 (8.7)	69795 (9.0)	0.010
COVID-19 vaccine	98079 (10.9)	70728 (4.8)	0.229	57346 (7.4)	52286 (6.8)	0.025
GLP-1 analogues	75944 (8.4)	70843 (4.8)	0.147	56172 (7.3)	56079 (7.2)	0.000
SGLT2 inhibitors	69962 (7.7)	73661 (5.0)	0.115	53358 (6.9)	53931 (7.0)	0.003
Laboratory data						
Glucose <70 mg/dL	53245 (5.9)	36280 (2.4)	0.174	33402 (4.3)	32009 (4.1)	0.009
Glucose ≥180 mg/dL	383625 (42.5)	349215 (23.5)	0.413	289201 (37.4)	288077 (37.2)	0.003
eGFR≥60 mL/min/1.73 m²	652239 (72.2)	737423 (49.6)	0.477	532056 (68.8)	536811 (69.4)	0.013
Hemoglobin ≥12 g/dL	627057 (69.5)	650939 (43.8)	0.536	504140 (65.2)	507930 (65.6)	0.010
Albumin ≥3.5 g/dL	591419 (65.5)	605873 (40.8)	0.512	471862 (61.0)	473947 (61.3)	0.006
HbA1c≥9%	135633 (15.0)	149644 (10.1)	0.150	108122 (14.0)	109122 (14.1)	0.004
Baseline ophthalmic surveillance						
Ophthalmology Services and Procedures	42950 (4.8)	43066 (2.9)	0.097	32366 (4.2)	32187 (4.2)	0.001

Data are presented as number (%) or mean ± standard deviation. Propensity score matching was performed at a 1:1 ratio using a greedy nearest-neighbor algorithm with a caliper of 0.1 pooled standard deviations of the propensity score logit. Standardized mean differences <0.10 were considered to indicate acceptable covariate balance. The detailed definition and included procedure concepts for Ophthalmology Services and Procedures are provided in **Supplementary Table 3**. BMI, body mass index; CKD, chronic kidney disease; COVID-19, coronavirus disease 2019; DPP-4, dipeptidyl peptidase-4; eGFR, estimated glomerular filtration rate; GLP-1, glucagon-like peptide-1; HbA1c, hemoglobin A1c; SGLT2, sodium-glucose cotransporter 2; SMD, standardized mean difference; T2DM, type 2 diabetes mellitus.

### 3.2 Primary and secondary outcomes

After propensity score matching, the mean follow-up time was 1,256.3 days in the COVID-19 group and 1,367.9 days in the control group, corresponding to approximately 3.44 and 3.75 years, respectively. The median follow-up time was 1,487 days in the COVID-19 group and 1,634 days in the control group. During follow-up within the maximum 5-year analytic window, overall DR occurred more often in the COVID-19 group than in the control group (2.17% vs. 1.64%; absolute risk difference, 0.53 percentage points) and was associated with a higher risk (HR, 1.51; 95% CI, 1.48–1.55) (**Table 2**). The E-value was 2.39 for the estimated hazard ratio and 2.32 for the lower limit of the 95% confidence interval. These values were considered a sensitivity measure for unmeasured confounding and were not interpreted as excluding residual confounding, including that related to diabetes duration.

**Table 2:** Association between COVID-19 and risks of diabetic retinopathy and related outcomes after propensity score matching.

Outcome	COVID-19 group (n=773,768)	Control group (n=773,768)	HR (95% CI)	*p* value	Absolute risk difference, percentage points
	Events (%)	Events (%)			
Primary outcome					
Overall DR	16,793 (2.17)	12,694 (1.64)	1.51 (1.48–1.55)	<0.001	+0.53
Secondary outcomes					
Non-proliferative DR	10,464 (1.35)	7,823 (1.01)	1.53 (1.49–1.58)	<0.001	+0.34
Proliferative DR	2,442 (0.32)	1,657 (0.21)	1.67 (1.57–1.78)	<0.001	+0.11
Diabetic macular edema	4,794 (0.62)	3,139 (0.41)	1.75 (1.67–1.83)	<0.001	+0.21
Hyperglycemia ≥180 mg/dL	315,110 (40.72)	273,925 (35.40)	1.35 (1.34–1.35)	<0.001	+5.32
HbA1c ≥9%	112,927 (14.59)	116,784 (15.09)	1.05 (1.04–1.05)	<0.001	-0.50
Hypoglycemia <70 mg/dL	65,868 (8.51)	42,474 (5.49)	1.75 (1.73–1.77)	<0.001	+3.02
Supportive outcomes					
Age-related cataract	48,049 (6.21)	49,226 (6.36)	1.06 (1.05–1.08)	<0.001	-0.15
Any healthcare visit	691,158 (89.32)	708,513 (91.57)	1.00 (1.00–1.00)	0.238	-2.25
Mortality	56,758 (7.34)	39,720 (5.13)	1.59 (1.57–1.61)	<0.001	+2.21

Data are presented as number of events (%). Hazard ratios were estimated using Cox proportional hazards models. Cataract was assessed as an ocular comparator outcome to evaluate nonspecific ophthalmic diagnostic activity, whereas any healthcare visit was assessed as a general healthcare-utilization marker and was interpreted descriptively because of its high frequency during follow-up. CI, confidence interval; COVID-19, coronavirus disease 2019; DR, diabetic retinopathy; HbA1c, hemoglobin A1c; HR, hazard ratio. Absolute risk differences are presented as percentage-point differences between groups.

Similar patterns were observed across DR subtypes (**Table 2**). Compared with the control group, the COVID-19 group showed higher risks of non-proliferative DR (1.35% vs. 1.01%; absolute risk difference, 0.34 percentage points; HR, 1.53; 95% CI, 1.49–1.58), proliferative DR (0.32% vs. 0.21%; absolute risk difference, 0.11 percentage points; HR, 1.67; 95% CI, 1.57–1.78), and diabetic macular edema (0.62% vs. 0.41%; absolute risk difference, 0.21 percentage points; HR, 1.75; 95% CI, 1.67–1.83). Metabolic outcomes showed similar patterns between groups. Hyperglycemia was associated with an HR of 1.35 (95% CI, 1.34–1.35), and hypoglycemia was associated with an HR of 1.75 (95% CI, 1.73–1.77). Although the proportion of patients with HbA1c ≥9% was slightly lower in the COVID-19 group (14.59% vs. 15.09%), time-to-event analysis showed a small positive association (HR, 1.05; 95% CI, 1.04–1.05). This apparent discrepancy likely reflects differences between crude end-of-follow-up event proportions and Cox time-to-event estimates, which account for event timing and censoring during follow-up. Mortality was also higher in the COVID-19 group (HR, 1.59; 95% CI, 1.57–1.61). Age-related cataract showed only a modest association (HR, 1.06; 95% CI, 1.05–1.08), whereas any healthcare visit showed no meaningful difference in time-to-event analysis (HR, 1.00; 95% CI, 1.00–1.00).

### 3.3 Sensitivity, subgroup, and multivariable analyses

The association between COVID-19 and DR remained consistent across sensitivity analyses (**Table 3**). Cohort sizes and event counts for each separately matched sensitivity and landmark analytic cohort are provided in **Supplementary Table 5**. For overall DR, the HR was 1.51 (95% CI, 1.47–1.55) in patients who survived during follow-up, 1.51 (95% CI, 1.47–1.54) among patients with at least one healthcare visit, 1.58 (95% CI, 1.54–1.62) in the 1-year landmark analysis, and 1.92 (95% CI, 1.86–1.98) in the 2-year landmark analysis. Comparable patterns were observed for non-proliferative DR, proliferative DR, diabetic macular edema, hyperglycemia, HbA1c ≥9%, and hypoglycemia. In the additional sensitivity analysis restricted to patients with at least one eye-care visit during follow-up, COVID-19 remained associated with a higher risk of overall DR (HR, 1.54; 95% CI, 1.47–1.60), non-proliferative DR (HR, 1.55; 95% CI, 1.48–1.63), proliferative DR (HR, 1.68; 95% CI, 1.50–1.87), and diabetic macular edema (HR, 1.67; 95% CI, 1.56–1.80) (**Supplementary Table 6**).

**Table 3:** Sensitivity analyses for the association between COVID-19 and study outcomes.

Outcomes	Model I		Model II		Model III		Model IV	
	HR (95% CI)	*p* value	HR (95% CI)	*p* value	HR (95% CI)	*p* value	HR (95% CI)	*p* value
Overall DR	1.51 (1.47–1.55)	<0.001	1.51 (1.47–1.54)	<0.001	1.58 (1.54–1.62)	<0.001	1.92 (1.86–1.98)	<0.001
Non-proliferative DR	1.52 (1.48–1.57)	<0.001	1.51 (1.47–1.56)	<0.001	1.59 (1.54–1.65)	<0.001	1.97 (1.89–2.04)	<0.001
Proliferative DR	1.63 (1.53–1.74)	<0.001	1.68 (1.57–1.79)	<0.001	1.65 (1.54–1.76)	<0.001	1.89 (1.74–2.06)	<0.001
Diabetic macular edema	1.70 (1.62–1.78)	<0.001	1.72 (1.65–1.80)	<0.001	1.77 (1.69–1.86)	<0.001	2.11 (1.99–2.24)	<0.001
Hyperglycemia ≥180 mg/dL	1.33 (1.32–1.33)	<0.001	1.34 (1.33–1.35)	<0.001	1.32 (1.31–1.32)	<0.001	1.34 (1.34–1.35)	<0.001
HbA1c ≥9%	1.04 (1.03–1.05)	<0.001	1.03 (1.02–1.04)	<0.001	1.04 (1.03–1.05)	<0.001	1.05 (1.04–1.06)	<0.001
Hypoglycemia <70 mg/dL	1.71 (1.69–1.73)	<0.001	1.74 (1.72–1.76)	<0.001	1.70 (1.68–1.72)	<0.001	1.75 (1.73–1.77)	<0.001

Model I included only patients who survived during follow-up. Model II included only patients with at least one healthcare visit during follow-up. Model III used a 1-year landmark design. Model IV used a 2-year landmark design. Propensity score matching was performed separately within each sensitivity and landmark analytic cohort. Hazard ratios were estimated using Cox proportional hazards models. CI, confidence interval; COVID-19, coronavirus disease 2019; DR, diabetic retinopathy; HbA1c, hemoglobin A1c; HR, hazard ratio.

In subgroup analyses (**Table 4**), the association with overall DR was observed across all prespecified strata. The HRs were 1.54 in men and 1.46 in women, with evidence of interaction by sex (p for interaction=0.020). Similar associations were observed in patients aged 18–65 years and >65 years, without significant interaction by age. Numerically higher HRs were observed in patients with chronic kidney disease, hypertension, HbA1c ≥9%, and hyperlipidemia, with statistically significant interactions for these factors. However, given the very large sample size, these interaction findings should be interpreted cautiously and may represent small differences of uncertain clinical relevance. No meaningful interaction was observed by obesity or smoking history. In the multivariable Cox model fitted independently of propensity score matching (**Table 5**), COVID-19 remained associated with higher risk of DR within the analytic window (HR, 1.56; 95% CI, 1.53–1.59).

**Table 4:** Subgroup analyses for the association between COVID-19 and incident diabetic retinopathy.

Subgroup (number of patients per group)	COVID group Events (%)	Control group Event (%)	HR (95% CI)	P-value	P for interaction
Sex					
Male (n=391,401)	8,541 (2.18)	6,437 (1.65)	1.54 (1.49–1.59)	<0.001	reference
Female (n=372,788)	7,909 (2.12)	6,125 (1.64)	1.46 (1.42–1.51)	<0.001	0.02
Age (years)					
18-65 (n=314,838)	7,505 (2.38)	5,278 (1.68)	1.55 (1.50–1.61)	<0.001	reference
>65 (n=454,159)	9,204 (2.03)	7,272 (1.60)	1.49 (1.44–1.54)	<0.001	0.114
CKD					
yes (n=96,662)	2,615 (2.71)	1,854 (1.92)	1.65 (1.56–1.76)	<0.001	reference
no (n=672,888)	14,017 (2.08)	10,840 (1.61)	1.47 (1.43–1.50)	<0.001	0.001
HTN					
yes (n=498,037)	12,204 (2.45)	8,668 (1.74)	1.60 (1.55–1.64)	<0.001	reference
no (n=257,363)	4,313 (1.68)	3,546 (1.38)	1.35 (1.29–1.41)	<0.001	<0.001
HbA1c ≥ 9%					
yes (n=125,608)	6,654 (5.30)	4,297 (3.42)	1.75 (1.68–1.81)	<0.001	reference
no (n=639,284)	9,790 (1.53)	7,911 (1.24)	1.41 (1.37–1.45)	<0.001	<0.001
Hyperlipidemia history					
yes (n=438,949)	11,354 (2.59)	7,927 (1.81)	1.62 (1.58–1.67)	<0.001	reference
no (n=314,855)	5,069 (1.61)	4,035 (1.28)	1.39 (1.33–1.45)	<0.001	<0.001
Obesity					
yes (n=251,261)	6,339 (2.52)	4,420 (1.76)	1.56 (1.50–1.62)	<0.001	reference
no (n=513,996)	10,342 (2.01)	8,019 (1.56)	1.50 (1.46–1.55)	<0.001	0.117
Smoking history					
yes (n=100,560)	2,114 (2.10)	1,530 (1.52)	1.53 (1.44–1.64)	<0.001	reference
no (n=669,801)	14,510 (2.17)	10,988 (1.64)	1.51 (1.48–1.55)	<0.001	0.711

Subgroup analyses were performed for the primary outcome of incident diabetic retinopathy. Hazard ratios were estimated using Cox proportional hazards models within each prespecified subgroup. P values for interaction were used to evaluate whether the association between COVID-19 and diabetic retinopathy differed across subgroup strata. CI, confidence interval; CKD, chronic kidney disease; COVID-19, coronavirus disease 2019; DR, diabetic retinopathy; HbA1c, hemoglobin A1c; HR, hazard ratio; HTN, hypertension.

**Table 5:** Multivariable Cox regression analysis for incident diabetic retinopathy.

Variables	HR (95% CI)	P-value
COVID-19 vs. Control groups	1.56 (1.53–1.59)	<0.001
Male	1.05 (1.03–1.07)	<0.001
Age at index	1.00 (1.00–1.00)	<0.001
White	0.90 (0.88–0.92)	<0.001
T2DM with kidney complications	1.52 (1.46–1.58)	<0.001
T2DM with neurological complications	1.96 (1.91–2.02)	<0.001
T2DM with circulatory complications	1.32 (1.27–1.38)	<0.001
Essential hypertension	0.94 (0.92–0.97)	<0.001
Disorders of lipoprotein metabolism and other lipidemias	0.97 (0.95–0.99)	0.008
Overweight and obesity	0.91 (0.89–0.93)	<0.001
Ischemic heart diseases	0.96 (0.94–0.99)	0.013
Cerebrovascular diseases	0.99 (0.96–1.03)	0.649
Heart failure	1.06 (1.01–1.10)	0.010
Chronic kidney disease	0.89 (0.86–0.93)	<0.001
Other chronic obstructive pulmonary disease	0.74 (0.71–0.78)	<0.001
Diseases of liver	0.86 (0.83–0.89)	<0.001
Other anemias	1.01 (0.97–1.04)	0.721
Malnutrition	0.70 (0.62–0.79)	<0.001
Nicotine dependence	0.89 (0.86–0.92)	<0.001
Neoplasms	0.86 (0.84–0.88)	<0.001
Alcohol-related disorders	0.84 (0.78–0.90)	<0.001

CI, confidence interval; COVID-19, coronavirus disease 2019; DR, diabetic retinopathy; HR, hazard ratio; T2DM, type 2 diabetes mellitus. This model was intended as a supportive robustness analysis of the COVID-19 association rather than as an etiologic risk-factor model; individual covariate coefficients should not be interpreted as independent causal, protective, or risk-factor estimates.

### 3.4 Severe-COVID, late-period, and competing-risk analyses

In the supportive severe-COVID analysis (**Table 6**), severe COVID-19 was associated with higher risks of overall DR (HR, 1.58; 95% CI, 1.53–1.63), non-proliferative DR (HR, 1.55; 95% CI, 1.48–1.61), proliferative DR (HR, 1.86; 95% CI, 1.72–2.02), and diabetic macular edema (HR, 1.81; 95% CI, 1.71–1.93). Mortality was also higher in the severe COVID-19 group (HR, 1.90; 95% CI, 1.87–1.93).

**Table 6:** Supportive severe-COVID analysis comparing patients with severe COVID-19 and matched controls.

Outcome	Severe COVID-19 group (n=419,649)	Control group (n=419,649)	HR (95% CI)	*P* value
	Events (%)	Events (%)		
Overall DR	9,285 (2.21)	7,002 (1.67)	1.58 (1.53–1.63)	<0.001
Non-proliferative DR	5,542 (1.32)	4,282 (1.02)	1.55 (1.48–1.61)	<0.001
Proliferative DR	1,483 (0.35)	937 (0.22)	1.86 (1.72–2.02)	<0.001
Diabetic macular edema	2,652 (0.63)	1,741 (0.41)	1.81 (1.71–1.93)	<0.001
Hyperglycemia ≥180 mg/dL	174,159 (41.50)	154,048 (36.71)	1.38 (1.37–1.39)	<0.001
HbA1c ≥9%	55,460 (13.22)	60,864 (14.50)	1.01 (1.00–1.03)	0.028
Hypoglycemia <70 mg/dL	44,744 (10.66)	26,863 (6.40)	1.97 (1.94–2.00)	<0.001
Age-related cataract	27,114 (6.46)	26,369 (6.28)	1.16 (1.14–1.18)	<0.001
Any healthcare visit	368,840 (87.89)	382,541 (91.16)	1.01 (1.01–1.02)	<0.001
Mortality	43,776 (10.43)	26,663 (6.35)	1.90 (1.87–1.93)	<0.001

Data are presented as number of events (%). Severe COVID-19 was defined as a COVID-19 index event with concurrent hospitalization or intensive care unit admission coding on the index date. This supportive analysis was not designed as a formal dose-response or exposure-gradient analysis. CI, confidence interval; COVID-19, coronavirus disease 2019; DR, diabetic retinopathy; HbA1c, hemoglobin A1c; HR, hazard ratio; ICU, intensive care unit.

**Figure 2:**
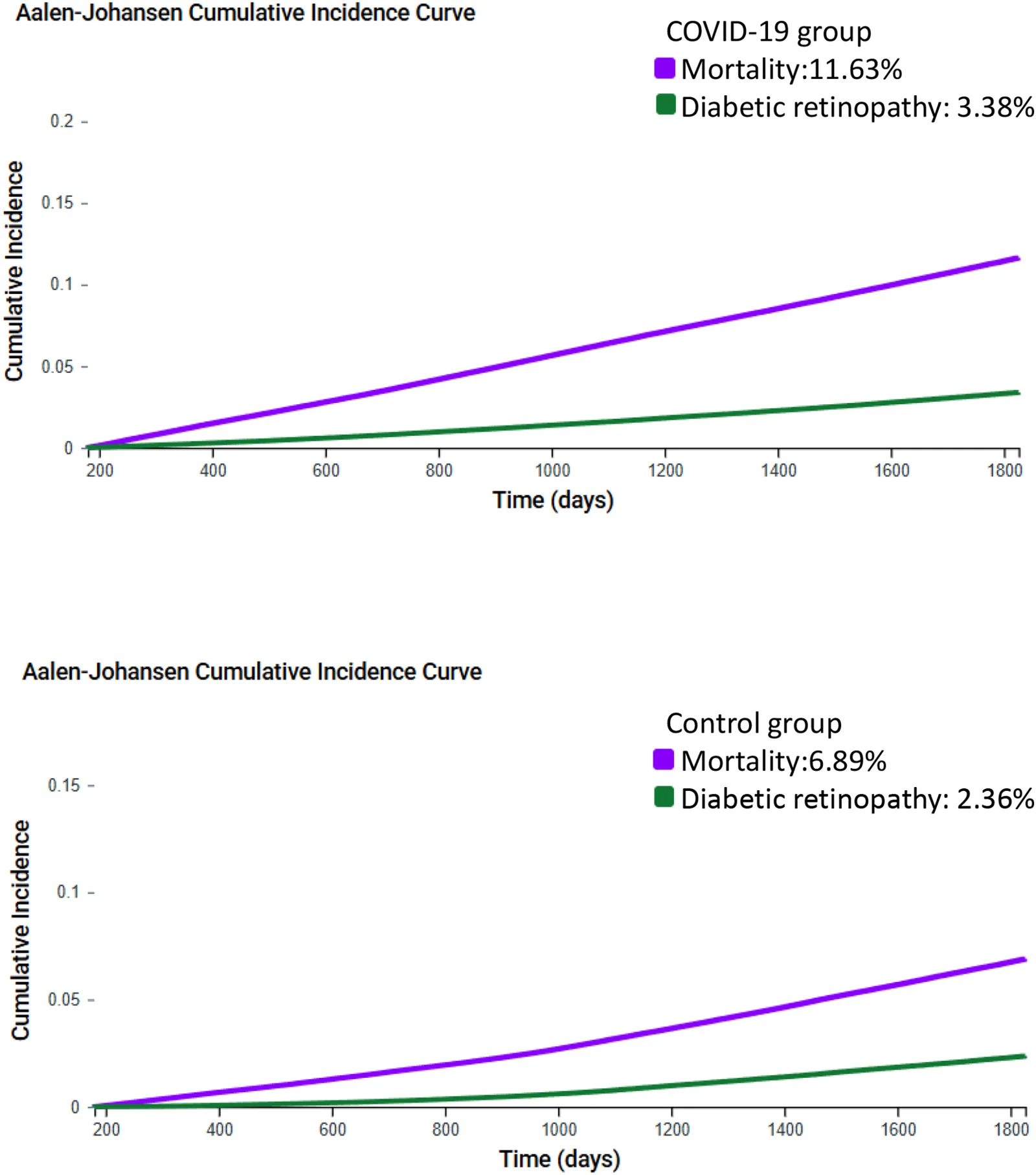
Unmatched descriptive Aalen–Johansen cumulative incidence curves for diabetic retinopathy with mortality as a competing event. Data were obtained from an unmatched descriptive competing-risk analysis. Mortality was modeled as a competing event, and diabetic retinopathy was the event of interest. Cumulative incidence curves were estimated using the Aalen–Johansen method and are presented over follow-up time. This supplemental analysis was performed in the unmatched aggregate cohort because matched-cohort competing-risk curves and number-at-risk tables were not available within the TriNetX workflow. DR, diabetic retinopathy.

In the late-period analysis restricted to first SARS-CoV-2 infection between July 2022 and June 2023, 136,631 patients were included in each group after matching (**Table 7**). The mean follow-up time was 881.6 days in the late COVID-19 group and 973.7 days in controls, and the median follow-up time was 1,067 days and 1,128 days, respectively. During available follow-up, positive associations remained for overall DR (HR, 1.33), non-proliferative DR (HR, 1.32), proliferative DR (HR, 1.45), and diabetic macular edema (HR, 1.42). In the supplemental descriptive competing-risk analysis conducted in the unmatched cohort, mortality was treated as a competing event. Aalen–Johansen cumulative incidence curves showed numerically higher cumulative incidence of DR in the COVID-19 group than in the control group, with end-of-follow-up cumulative incidence of 3.38% and 2.36%, respectively. Mortality was also numerically higher in the COVID-19 group than in the control group (11.63% vs. 6.89%) (**Figure 2**).

**Table 7:** Late-period analysis comparing patients with first SARS-CoV-2 infection between July 2022 and June 2023 with matched controls.

Outcome	Late COVID-19 group (n=136,631)	Late control group (n=136,631)	HR (95% CI)	*P* value
	Events (%)	Events (%)		
Overall DR	2,550 (1.87)	2,220 (1.62)	1.33 (1.25–1.40)	<0.001
Non-proliferative DR	1,591 (1.16)	1,395 (1.02)	1.32 (1.23–1.42)	<0.001
Proliferative DR	371 (0.27)	297 (0.22)	1.45 (1.24–1.69)	<0.001
Diabetic macular edema	732 (0.54)	597 (0.44)	1.42 (1.28–1.59)	<0.001
Hyperglycemia ≥180 mg/dL	46,607 (34.11)	44,141 (32.31)	1.20 (1.18–1.21)	<0.001
HbA1c ≥9%	14,834 (10.86)	16,145 (11.82)	1.01 (0.99–1.04)	0.252
Hypoglycemia <70 mg/dL	8,008 (5.86)	7,303 (5.35)	1.24 (1.20–1.28)	<0.001
Age-related cataract	6,956 (5.09)	7,179 (5.25)	1.08 (1.04–1.11)	<0.001
Any healthcare visit	119,334 (87.34)	125,743 (92.03)	0.91 (0.90–0.92)	<0.001
Mortality	7,990 (5.85)	5,778 (4.23)	1.57 (1.52–1.62)	<0.001

Data are presented as number of events (%). This exploratory analysis was restricted to patients with first recorded COVID-19 infection from July 2022 to June 2023. The control group included matched patients with type 2 diabetes who had no recorded history of COVID-19. This analysis was performed to evaluate whether the association between COVID-19 and diabetic retinopathy persisted during the later pandemic period. CI, confidence interval; COVID-19, coronavirus disease 2019; DR, diabetic retinopathy; HbA1c, hemoglobin A1c; HR, hazard ratio; T2DM, type 2 diabetes mellitus.

## 4 Discussion

In this large multinational propensity score–matched cohort of adults with type 2 diabetes mellitus, COVID-19 was associated with a higher risk of EHR-recorded incident DR during follow-up within a maximum 5-year analytic window. This association was observed for overall DR and for clinically important subtypes, including proliferative DR and diabetic macular edema. The findings were generally consistent across sensitivity analyses, subgroup analyses, an unmatched multivariable model, and analyses of severe and later-period COVID-19 infection. The observed associations remained after adjustment for multiple baseline clinical factors and were not accompanied by a substantial increase in overall healthcare visits. Importantly, the association also persisted in an additional sensitivity analysis restricted to patients with at least one eye-care visit during follow-up, supporting the robustness of the finding after partially accounting for differential ophthalmic ascertainment. Taken together, these findings identify an association between COVID-19 and EHR-recorded DR diagnoses among patients with type 2 diabetes, rather than establishing biologically confirmed DR progression or causality.

Direct evidence on incident DR after COVID-19 remains limited. The main available cohort study (19) was conducted within a single integrated health system and used electronic health record data to compare patients with T2DM who developed COVID-19 with contemporaneous controls without documented infection. Using propensity score matching and time-to-event analyses over approximately three years of follow-up, the authors (19) reported an increased risk of newly diagnosed DR after COVID-19, with an adjusted HR of approximately 1.24, although detailed analyses of DR severity and specific subtypes were not performed. The study (19) provided important preliminary evidence but was limited by its single-system setting and relatively shorter follow-up duration. Our study expands on these findings by using a large multinational federated network, allowing follow-up for up to five years, and evaluating clinically relevant DR subtypes, including proliferative DR and diabetic macular edema. The results are also consistent with broader evidence linking COVID-19 to post-acute cardiometabolic and vascular sequelae (21–23), suggesting that retinal microvascular outcomes may represent part of the longer-term clinical burden observed after SARS-CoV-2 infection.

A clinically important finding was that the association between COVID-19 and incident DR did not appear limited to the early post-infection period. In the primary 6-month landmark analysis, COVID-19 was associated with a higher risk of overall DR (HR, 1.51). When early events were further excluded using 1-year and 2-year landmark analyses, the association persisted, with HRs of 1.58 and 1.92, respectively. This persistence after excluding early events is reassuring, but it does not rule out residual detection or ascertainment bias. Longer landmarking may introduce selection effects, differential survival conditioning, delayed ophthalmic screening after pandemic-related disruptions, and depletion of susceptible individuals. Therefore, these analyses should be interpreted as showing that the association persisted after early event exclusion, rather than as evidence that short-term detection bias was eliminated. In a supportive analysis, patients with severe COVID-19, defined by a COVID-19 index event with concurrent hospitalization or intensive care unit admission coding, showed positive associations with overall DR (HR, 1.58), proliferative DR (HR, 1.86), and diabetic macular edema (HR, 1.81). However, these findings should be interpreted cautiously because hospitalization and intensive care unit admission may reflect not only COVID-19 severity but also baseline frailty, inpatient metabolic stress, systemic inflammation, treatment exposure, healthcare intensity, and follow-up opportunity. This analysis was not a formal dose–response or interaction analysis comparing severity strata; therefore, we avoided interpreting it as definitive evidence of an exposure-gradient pattern.

Another important feature of the findings was the subtype pattern of DR risk. The association was not confined to overall or non-proliferative DR but was also observed for proliferative DR and diabetic macular edema, two outcomes more closely linked to vision-threatening diabetic eye disease (24, 25). In the primary analysis, the relative association was numerically stronger for proliferative DR (HR, 1.67) and diabetic macular edema (HR, 1.75) than for non-proliferative DR (HR, 1.53). This pattern is clinically relevant because advanced DR subtypes are more directly related to the need for ophthalmic treatment and visual prognosis. However, these findings should not be interpreted as excluding detection bias. Although baseline ophthalmic surveillance was balanced after matching and the association persisted in an eye-care-visit-restricted sensitivity analysis, residual differences in retinal imaging frequency, ophthalmology or optometry documentation, increased follow-up eye-care intensity after COVID-19, and coding practices may still have influenced DR detection, documentation, and coding. Therefore, the subtype findings should be interpreted as associations with coded clinical diagnoses rather than as definitive evidence of biological DR progression.

Several supportive analyses helped contextualize, but do not eliminate, potential sources of bias. Age-related cataract, included as an ocular comparator outcome, showed only a modest association with COVID-19 (HR, 1.06), and overall healthcare visits were similar between groups in time-to-event analysis (HR, 1.00). In addition, the association between COVID-19 and DR persisted in a sensitivity analysis restricted to patients with at least one eye-care visit during follow-up. These findings suggest that the observed DR association was not accompanied by a broad increase in ocular diagnoses or general healthcare utilization, although more specific differences in ophthalmic examination frequency could not be fully assessed. In the late-period analysis restricted to first SARS-CoV-2 infection during 2022–2023, the association with overall DR was attenuated but remained present (HR, 1.33), suggesting that the finding was not limited to the earliest pandemic period. Nevertheless, residual confounding related to differences in index-date distribution and pandemic-era changes in healthcare access, diagnostic practices, vaccination, and COVID-19 management cannot be completely excluded. In the supplemental descriptive competing-risk analysis conducted in the unmatched cohort, cumulative incidence of DR was numerically higher in the COVID-19 group despite higher mortality. Because this analysis was unmatched and descriptive, it should not be interpreted as validating the matched cause-specific Cox estimates. Accordingly, the matched cause-specific hazard ratios should be interpreted as relative hazards among patients remaining at risk rather than as estimates of the cumulative probability of developing DR in the presence of competing mortality.

In the current study, the association with poor chronic glycemic control, defined by HbA1c ≥9%, was small. This pattern suggests that the higher risk of DR may not be explained solely by a sustained worsening of long-term glycemic control. Because HbA1c was evaluated as a categorical threshold-defined variable rather than using longitudinal HbA1c trajectories or time-updated glycemic measurements, this finding should be interpreted cautiously. Additional biological mechanisms may also be relevant, including post-acute endothelial dysfunction, inflammatory activation, immunothrombosis, and microvascular injury, which may contribute to retinal microvascular vulnerability after SARS-CoV-2 infection (7–10). Episodic dysglycemia or glycemic variability may also contribute, but the available data do not allow these mechanisms to be separated. Because the TriNetX platform does not support formal mediation analysis and laboratory testing was not uniformly available, these biological explanations remain hypotheses and should not be interpreted as mechanisms demonstrated by the present study.

These findings may have practical implications for post-pandemic diabetes care. Given the large number of patients with T2DM who have recovered from COVID-19, even a modest increase in DR risk could translate into a meaningful population-level burden. A history of COVID-19 may be considered a potential risk marker, rather than a proven causal risk factor, when assessing retinal risk in patients with T2DM. Although numerically stronger associations were observed among patients with chronic kidney disease, hypertension, poor glycemic control, and hyperlipidemia, these exploratory subgroup differences were modest and of uncertain clinical relevance. However, because the absolute event rates were modest and the study was observational, the current evidence is insufficient to support changes in DR screening intervals based solely on COVID-19 history. Prospective studies are needed to determine whether post-COVID retinal surveillance strategies improve outcomes.

This study has several limitations. First, the analysis relied on de-identified electronic health records, and both COVID-19 exposure and DR outcomes were identified using coded diagnoses, laboratory records, and available clinical data. Misclassification is therefore possible, particularly for asymptomatic or home-tested SARS-CoV-2 infections and for retinal disease managed outside participating organizations. Because controls were defined by no recorded COVID-19 in the available EHR, undocumented infections, particularly after widespread home testing, may have caused exposure misclassification. In addition, controls who later developed recorded COVID-19 were retained according to their baseline exposure classification, which may have introduced exposure crossover. Such exposure crossover and misclassification could attenuate or otherwise affect the observed association, although the exact direction and magnitude of bias cannot be determined. Because empirically supported exposure-misclassification probabilities were not available from the aggregate TriNetX data, we did not perform a quantitative bias correction based on assumed misclassification rates. Second, although propensity score matching and multivariable adjustment included a broad range of demographic, systemic, metabolic, ophthalmic, and medication-related covariates, residual confounding remains possible. Differential availability of laboratory measurements may have affected the classification of laboratory covariates used for propensity score matching and the ascertainment of laboratory-based secondary outcomes, particularly if testing was related to disease severity or healthcare utilization. Diabetes-related complications, insulin use, and glycemic markers were included and may partially capture differences in diabetes chronicity and severity. Important factors such as diabetes duration, baseline DR screening status, insurance status, access to ophthalmic care, socioeconomic status, HbA1c trajectories, follow-up eye-care intensity, retinal imaging frequency, visual acuity, and detailed ophthalmic examination findings were not uniformly available. Therefore, the outcome should be interpreted as EHR-recorded incident DR rather than adjudicated biologically confirmed incident DR. Third, DR severity was based on diagnostic codes rather than standardized fundus photography or adjudicated retinal grading, which may introduce outcome misclassification and limit assessment of functional visual outcomes. Post-index treatment procedure codes, including intravitreal injection, retinal laser, or vitrectomy, were not evaluated as supportive markers because these aggregate codes may reflect non-DR retinal indications and may be influenced by treatment access, referral patterns, physician practice, and timing relative to the coded DR diagnosis. The retinal endpoints should therefore be interpreted as EHR-recorded coded clinical diagnoses rather than standardized retinal imaging-confirmed incident DR. The low absolute event rates suggest likely under-ascertainment of coded incident DR in this large T2DM cohort. Fourth, subgroup, sensitivity, severe-COVID, late-period, and competing-risk analyses were exploratory and should be interpreted as hypothesis-generating. Finally, although the federated network included 177 participating healthcare organizations, organization-level geographic distribution was not available in the aggregate output, limiting assessment of the cohort’s representativeness across specific regions and healthcare systems. Prospective studies using standardized retinal imaging and longer follow-up are needed to confirm these findings and clarify their clinical implications.

## 5 Conclusion

In this large multicenter EHR-based propensity score–matched cohort of adults with T2DM, recorded SARS-CoV-2 infection was associated with a higher subsequent risk of coded incident DR, including proliferative DR and diabetic macular edema, during follow-up within a maximum 5-year analytic window. Because outcomes were identified from routine clinical coding and residual confounding, incomplete exposure capture, differential ophthalmic surveillance, and competing mortality remain possible, these findings should be interpreted as hypothesis-generating. Independent confirmation in prospective cohorts with patient-level data and standardized retinal imaging is warranted.

## Data Availability

The datasets presented in this article are not readily available because The datasets analyzed in this study were obtained from the TriNetX Global Collaborative Network and are subject to TriNetX data-use agreements and institutional restrictions. The authors do not have access to identifiable individual-level patient data and are not permitted to publicly share or redistribute the underlying datasets. Access to the data may be available only through TriNetX for users with appropriate authorization and in accordance with applicable data-use agreements. Requests to access the datasets should be directed to https://live.trinetx.com.

## References

[1] Dulull N Kwa F Osman N Rai U Shaikh B Thrimawithana TR . Recent advances in the management of diabetic retinopathy. Drug Discov Today. (2019) 24:1499–509. doi: 10.1016/j.drudis.2019.03.028 30954684

[2] Faselis C Katsimardou A Imprialos K Deligkaris P Kallistratos M Dimitriadis K . Microvascular complications of type 2 diabetes mellitus. Curr Vasc Pharmacol. (2020) 18:117–24. doi: 10.2174/1570161117666190502103733 31057114

[3] Simó-Servat O Hernández C Simó R . Diabetic retinopathy in the context of patients with diabetes. Ophthalmic Res. (2019) 62:211–7. doi: 10.1159/000499541 31129667

[4] Teo ZL Tham YC Yu M Chee ML Rim TH Cheung N . Global prevalence of diabetic retinopathy and projection of burden through 2045: Systematic review and meta-analysis. Ophthalmology. (2021) 128:1580–91. doi: 10.1016/j.ophtha.2021.04.027 33940045

[5] American Diabetes Association Professional Practice Committee for Diabetes . Retinopathy, neuropathy, and foot care: Standards of care in diabetes-2026. Diabetes Care. (2026) 49:S261–76. doi: 10.2337/9781580408462-ch19 PMC12690177 41358886

[6] Lim JI Kim SJ Bailey ST Kovach JL Vemulakonda GA Ying GS . Diabetic retinopathy preferred practice pattern®. Ophthalmology. (2025) 132:P75–P162. doi: 10.1016/j.ophtha.2019.09.025 39918521

[7] Nalbandian A Sehgal K Gupta A Madhavan MV McGroder C Stevens JS . Post-acute COVID-19 syndrome. Nat Med. (2021) 27:601–15. doi: 10.1038/s41591-021-01283-z 33753937 PMC8893149

[8] Xie Y Xu E Bowe B Al-Aly Z . Long-term cardiovascular outcomes of COVID-19. Nat Med. (2022) 28:583–90. doi: 10.1038/s41591-022-01689-3 35132265 PMC8938267

[9] Bonaventura A Vecchié A Dagna L Martinod K Dixon DL Van Tassell BW . Endothelial dysfunction and immunothrombosis as key pathogenic mechanisms in COVID-19. Nat Rev Immunol. (2021) 21:319–29. doi: 10.1038/s41577-021-00536-9 33824483 PMC8023349

[10] Xie Y Al-Aly Z . Risks and burdens of incident diabetes in long COVID: a cohort study. Lancet Diabetes Endocrinol. (2022) 10:311–21. doi: 10.1016/s2213-8587(22)00044-4 35325624 PMC8937253

[11] Xiong X Lui DTW Chung MSH Au ICH Lai FTT Wan EYF . Incidence of diabetes following COVID-19 vaccination and SARS-CoV-2 infection in Hong Kong: A population-based cohort study. PloS Med. (2023) 20:e1004274. doi: 10.1371/journal.pmed.1004274 37486927 PMC10406181

[12] Taylor K Eastwood S Walker V Cezard G Knight R Al Arab M . Incidence of diabetes after SARS-CoV-2 infection in England and the implications of COVID-19 vaccination: a retrospective cohort study of 16 million people. Lancet Diabetes Endocrinol. (2024) 12:558–68. doi: 10.1016/s2213-8587(24)00159-1 39054034 PMC7617111

[13] Ssentongo P Zhang Y Witmer L Chinchilli VM Ba DM . Association of COVID-19 with diabetes: a systematic review and meta-analysis. Sci Rep. (2022) 12:20191. doi: 10.1038/s41598-022-24185-7 36418912 PMC9684130

[14] Montefusco L Ben Nasr M D'Addio F Loretelli C Rossi A Pastore I . Acute and long-term disruption of glycometabolic control after SARS-CoV-2 infection. Nat Metab. (2021) 3:774–85. doi: 10.1038/s42255-021-00407-6 34035524 PMC9931026

[15] Lawal B Saidu Musa S Soe Thu M Ondee T Chatsirisakul O Pongpirul K . Post-acute metabolic changes and risk of new-onset diabetes following COVID-19: a systematic review and meta-analysis. Front Endocrinol (Lausanne). (2026) 17:1835180. doi: 10.3389/fendo.2026.1835180 42255427 PMC13233281

[16] Ahmed I Liu TYA . The impact of COVID-19 on diabetic retinopathy monitoring and treatment. Curr Diabetes Rep. (2021) 21:40. doi: 10.1007/s11892-021-01411-6 34495377 PMC8425316

[17] Pardhan S Wijewickrama RCA Gilbert CE Piyasena MP Sapkota R . Impact of COVID-19 and recovery of routine diabetic retinopathy digital screening across different regions in England: an analysis of publicly available data. BMJ Open. (2024) 14:e089710. doi: 10.1136/bmjopen-2024-089710 39732486 PMC11683968

[18] Scanlon PH Norridge CFE Prentis D Holman N Rankin P Valabhji J . Effect of the COVID-19 pandemic on diabetic retinopathy and referral levels in the English National Health Service Diabetic Eye Screening Programme. Diabetes Med. (2025) 42:e15518. doi: 10.1002/9781444324808.ch36 39901468 PMC12006553

[19] Mehrotra-Varma J Henry S Chernoff D Galenchik-Chan A Duong KS Mehrotra-Varma S . Increased incidence of new-onset diabetic retinopathy in individuals with COVID-19 in an underserved urban population in the Bronx. Diagn (Basel). (2025) 15(15):1846. doi: 10.3390/diagnostics15151846 40804810 PMC12345821

[20] Symes RJ Liew G Tufail A . Sight-threatening diabetic eye disease: an update and review of the literature. Br J Gen Pract. (2014) 64:e678-80. doi: 10.3399/bjgp14x682033 25267057 PMC4173734

[21] Raman B Bluemke DA Lüscher TF Neubauer S . Long COVID: post-acute sequelae of COVID-19 with a cardiovascular focus. Eur Heart J. (2022) 43:1157–72. doi: 10.1093/eurheartj/ehac031 35176758 PMC8903393

[22] Kole C Stefanou E Karvelas N Schizas D Toutouzas KP . Acute and post-acute COVID-19 cardiovascular complications: a comprehensive review. Cardiovasc Drugs Ther. (2024) 38:1017–32. doi: 10.1007/s10557-023-07465-w 37209261 PMC10199303

[23] Sherif ZA Gomez CR Connors TJ Henrich TJ Reeves WB . Pathogenic mechanisms of post-acute sequelae of SARS-CoV-2 infection (PASC). Elife. (2023) 12:e86002. doi: 10.7554/elife.86002 36947108 PMC10032659

[24] Lee R Wong TY Sabanayagam C . Epidemiology of diabetic retinopathy, diabetic macular edema and related vision loss. Eye Vis (Lond). (2015) 2:17. doi: 10.1186/s40662-015-0026-2 26605370 PMC4657234

[25] Khurana RN Wang JC Zhang S Li C Lum F . Outcomes of patients with proliferative diabetic retinopathy treated with anti-VEGF therapy and/or panretinal photocoagulation in the United States who were lost to follow-up. Ophthalmol Retina. (2025) 9:1167–74. doi: 10.1016/j.oret.2025.05.033 40473110

