# Supplementary material for: COVID-19 and subsequent risk of coded diabetic retinopathy in patients with type 2 diabetes: a multicenter propensity score-matched cohort study
Source: Front Endocrinol (Lausanne). 2026 Sep 11;17:1914361. doi: 10.3389/fendo.2026.1914361 (PMC13612189; doi:10.3389/fendo.2026.1914361)
Supplement: Supplementary file 1 [file Table1.docx]

**Supplemental Table 1. Codes used to define cohort eligibility, exposure, and exclusion criteria**

| Criterion | Code set / definition |
| --- | --- |
| Age eligibility | TriNetX demographic variable; age ≥18 years |
| Type 2 diabetes mellitus | ICD-10-CM E11 |
| SARS-CoV-2 infection | ICD-10-CM U07.1; TriNetX laboratory term TNX:9088, SARS coronavirus 2 and related RNA [Presence] |
| Absence of documented SARS-CoV-2 infection in control cohort | No ICD-10-CM U07.1 and no positive TNX:9088 SARS coronavirus 2 and related RNA [Presence] record |
| Baseline healthcare encounter requirement | TriNetX visit term: TNX:Visit |
| Pre-existing diabetic ocular disease | ICD-10-CM E11.3 |
| Early incident diabetic retinopathy during 6-month landmark period | ICD-10-CM E11.31, E11.32, E11.33, E11.34, E11.35 |
| Early death or uncertain mortality coding during 6-month landmark period | TriNetX deceased status; ICD-10-CM R99 |
| Type 1 diabetes mellitus | ICD-10-CM E10 |
| Gestational diabetes mellitus | ICD-10-CM O24.4 |
| Advanced chronic kidney disease | ICD-10-CM N18.4, N18.5 |
| End-stage renal disease or dialysis dependence | ICD-10-CM N18.6, Z99.2 |
| Bariatric surgery status | ICD-10-CM Z98.84 |
| Blindness or low vision | ICD-10-CM H54 |
| Other retinal disorders | ICD-10-CM H35 |
| Retinal vascular occlusion | ICD-10-CM H34 |
| Retinal detachment or retinal breaks | ICD-10-CM H33 |
| Prior intravitreal injection | CPT 67028 |
| Prior vitrectomy | CPT 67036, 67039, 67040, 67041, 67042, 67043 |
| Prior retinal or choroidal photocoagulation | CPT 67210, 67220 |
| Prior treatment for extensive or progressive retinopathy | CPT 67228 |

Unless otherwise specified, exclusion diagnoses and procedures were assessed before the index date. Early incident diabetic retinopathy and early death were assessed during the 6-month landmark period after the index date. ICD-10-CM parent-code prefixes were selected at the parent-concept level in the TriNetX hierarchy and included all available descendant codes, including laterality-specific, severity-specific, and eye-unspecified extensions when present.

**Supplemental Table 2. Availability and missingness of pre-index laboratory covariates after propensity score matching**

| Laboratory covariate | Available laboratory record, COVID-19 | Available laboratory record, Control | Missing laboratory record, COVID-19 | Missing laboratory record, Control | Threshold-defined covariate, COVID-19 | Threshold-defined covariate, Control | SMD |
| --- | --- | --- | --- | --- | --- | --- | --- |
| eGFR ≥60 mL/min/1.73 m² | 588,486 (76.05) | 581,902 (75.20) | 185,282 (23.95) | 191,866 (24.80) | 532,056 (68.76) | 536,811 (69.38) | 0.013 |
| Glucose ≥180 mg/dL | 579,271 (74.86) | 555,779 (71.83) | 194,497 (25.14) | 217,989 (28.17) | 289,201 (37.38) | 288,077 (37.23) | 0.003 |
| Glucose <70 mg/dL | 579,271 (74.86) | 555,779 (71.83) | 194,497 (25.14) | 217,989 (28.17) | 33,402 (4.32) | 32,009 (4.14) | 0.009 |
| Hemoglobin ≥12 g/dL | 568,861 (73.52) | 544,015 (70.31) | 204,907 (26.48) | 229,753 (29.69) | 504,140 (65.15) | 507,930 (65.64) | 0.010 |
| Albumin ≥3.5 g/dL | 503,915 (65.12) | 490,096 (63.34) | 269,853 (34.88) | 283,672 (36.66) | 471,862 (60.98) | 473,947 (61.25) | 0.006 |
| HbA1c ≥9% | 432,220 (55.86) | 441,729 (57.09) | 341,548 (44.14) | 332,039 (42.91) | 108,122 (13.97) | 109,122 (14.10) | 0.004 |

Data are presented as number (%). Laboratory availability was defined as having at least one recorded value for the corresponding test during the 5-year pre-index lookback period. Missing laboratory record was calculated using the matched cohort denominator of 773,768 patients per group. Threshold-defined covariates indicate patients with a recorded pre-index laboratory value meeting the prespecified threshold used in propensity score matching. Missing laboratory values were not imputed; therefore, absence of a recorded threshold-defined laboratory value should not be interpreted as confirmed normal physiology. eGFR, estimated glomerular filtration rate; HbA1c, hemoglobin A1c; SMD, standardized mean difference.

**Supplemental Table 3. Codes for variables used in propensity score matching**

| **Matching variable** | **Code set / definition** |
| --- | --- |
| Age at index | TriNetX demographic variable |
| Sex | TriNetX demographic variable |
| Body mass index >30 kg/m² | TriNetX BMI vital/laboratory record; BMI >30 kg/m² |
| White | TriNetX race variable |
| Black or African American | TriNetX race variable |
| Asian | TriNetX race variable |
| Essential hypertension | ICD-10-CM I10 |
| Disorders of lipoprotein metabolism and other lipidemias | ICD-10-CM E78 |
| Overweight and obesity | ICD-10-CM E66 |
| Neoplasms | ICD-10-CM C00–D49 |
| Ischemic heart diseases | ICD-10-CM I20–I25 |
| Nicotine dependence | ICD-10-CM F17 |
| Diseases of liver | ICD-10-CM K70–K77 |
| Chronic kidney disease | ICD-10-CM N18 |
| Cerebrovascular diseases | ICD-10-CM I60–I69 |
| Type 2 diabetes mellitus with neurological complications | ICD-10-CM E11.4 |
| Type 2 diabetes mellitus with kidney complications | ICD-10-CM E11.2 |
| Type 2 diabetes mellitus with circulatory complications | ICD-10-CM E11.5 |
| Heart failure | ICD-10-CM I50 |
| Other chronic obstructive pulmonary disease | ICD-10-CM J44 |
| Other peripheral vascular diseases | ICD-10-CM I73 |
| Age-related cataract | ICD-10-CM H25 |
| Glaucoma | ICD-10-CM H40–H42 |
| Alcohol-related disorders | ICD-10-CM F10 |
| Malnutrition | ICD-10-CM E40–E46 |
| Ophthalmology services and procedures | TriNetX procedure hierarchy term 1012793, Ophthalmology Services and Procedures, including available descendant CPT-based ophthalmology service/procedure concepts captured within the TriNetX platform. Descendant concepts included general ophthalmological services and procedures, new-patient and established-patient ophthalmological services, special ophthalmological services and procedures, ophthalmological examination/evaluation under anesthesia, visual field examination, ophthalmoscopy procedures, other specialized ophthalmological services and procedures, computerized ophthalmic diagnostic imaging including posterior-segment optical coherence tomography, orthoptic training, determination of refractive state, gonioscopy, computerized corneal topography, sensorimotor examination, contact lens services, serial tonometry, anterior-segment computerized ophthalmic diagnostic imaging, ophthalmic biometry, glaucoma provocative testing, corneal hysteresis determination, spectacle services, and other ophthalmological services or procedures. This variable was used as a baseline ophthalmic surveillance/utilization covariate rather than as a retinal disease outcome. |
| Antilipemic agents | TriNetX medication term CV350 / ATC C10 |
| Insulins and analogues | ATC A10A |
| Biguanides | ATC A10BA |
| Sulfonylureas | ATC A10BB |
| Dipeptidyl peptidase-4 inhibitors | ATC A10BH |
| Glucagon-like peptide-1 analogues | ATC A10BJ |
| Sodium-glucose cotransporter-2 inhibitors | ATC A10BK |
| SARS-CoV-2 mRNA vaccine | TriNetX medication term 2468231, SARS-CoV-2 (COVID-19) vaccine, mRNA spike protein |
| Glucose <70 mg/dL | TriNetX laboratory term TNX:9025; glucose ≤69 mg/dL |
| Glucose ≥180 mg/dL | TriNetX laboratory term TNX:9025; glucose ≥180 mg/dL |
| Estimated glomerular filtration rate ≥60 mL/min/1.73 m² | TriNetX laboratory term TNX:8001; eGFR ≥60 mL/min/1.73 m² |
| Hemoglobin ≥12 g/dL | TriNetX laboratory term TNX:9014; hemoglobin ≥12 g/dL |
| Albumin ≥3.5 g/dL | TriNetX laboratory term TNX:9045; albumin ≥3.5 g/dL |
| Hemoglobin A1c ≥9% | TriNetX laboratory term TNX:9037; HbA1c ≥9% |

All matching variables were assessed during the 5-year pre-index lookback period and were required to be recorded before the index date. For diagnosis and procedure concepts selected using parent-code prefixes or hierarchy terms, all available descendant concepts in the TriNetX hierarchy were included when present. Laboratory covariates were threshold-defined features based on recorded pre-index laboratory values and were not imputed when missing.

**Supplemental Table 4. Definitions of primary, secondary, and supportive outcomes used in the TriNetX analysis**

| Outcome | Code set / TriNetX term |
| --- | --- |
| Primary outcome |  |
| Overall diabetic retinopathy | ICD-10-CM E11.31, E11.32, E11.33, E11.34, E11.35 |
| Secondary outcomes |  |
| Non-proliferative diabetic retinopathy | ICD-10-CM E11.32, E11.33, E11.34 |
| Proliferative diabetic retinopathy | ICD-10-CM E11.35 |
| Diabetic macular edema | ICD-10-CM E11.311, E11.321, E11.331, E11.341, E11.351 |
| Hyperglycemia ≥180 mg/dL | TriNetX laboratory term TNX:9025, Glucose [Mass/volume] in serum, plasma, or blood ≥180 mg/dL |
| Poor glycemic control | TriNetX laboratory term TNX:9037, Hemoglobin A1c/Hemoglobin.total in blood ≥9.00% |
| Hypoglycemia <70 mg/dL | TriNetX laboratory term TNX:9025, Glucose [Mass/volume] in serum, plasma, or blood <70 mg/dL |
| Supportive outcomes |  |
| Age-related cataract | ICD-10-CM H25 |
| Any healthcare visit | TriNetX visit term TNX:Visit |
| All-cause mortality | TriNetX deceased status; ICD-10-CM R99 |

**Supplemental Table 5.** Cohort sizes and event counts for sensitivity and landmark analyses

| Model I | Outcome | COVID-19 group (n=721,026), Events (%) | Control group (n=721,026), Events (%) |
| --- | --- | --- | --- |
|  | Overall diabetic retinopathy | 15,933 (2.21) | 11,926 (1.65) |
|  | Non-proliferative DR | 10,031 (1.39) | 7,452 (1.03) |
|  | Proliferative DR | 2,300 (0.32) | 1,576 (0.22) |
|  | Diabetic macular edema | 4,548 (0.63) | 3,020 (0.42) |
|  | Hyperglycemia ≥180 mg/dL | 285,799 (39.64) | 249,832 (34.65) |
|  | HbA1c ≥9% | 107,126 (14.86) | 110,460 (15.32) |
|  | Hypoglycemia <70 mg/dL | 54,588 (7.57) | 35,632 (4.94) |
| Model II | Outcome | COVID-19 group (n=689,312), Events (%) | Control group (n=689,312), Events (%) |
|  | Overall diabetic retinopathy | 16,670 (2.42) | 12,391 (1.80) |
|  | Non-proliferative DR | 10,376 (1.51) | 7,686 (1.12) |
|  | Proliferative DR | 2,404 (0.35) | 1,591 (0.23) |
|  | Diabetic macular edema | 4,740 (0.69) | 3,083 (0.45) |
|  | Hyperglycemia ≥180 mg/dL | 314,316 (45.60) | 267,967 (38.88) |
|  | HbA1c ≥9% | 112,166 (16.27) | 114,605 (16.63) |
|  | Hypoglycemia <70 mg/dL | 65,585 (9.52) | 41,538 (6.03) |
| Model III | Outcome | COVID-19 group (n=760,239), Events (%) | Control group (n=760,239), Events (%) |
|  | Overall diabetic retinopathy | 14,627 (1.92) | 10,672 (1.40) |
|  | Non-proliferative DR | 9,074 (1.19) | 6,570 (0.86) |
|  | Proliferative DR | 2,064 (0.27) | 1,428 (0.19) |
|  | Diabetic macular edema | 4,156 (0.55) | 2,701 (0.36) |
|  | Hyperglycemia ≥180 mg/dL | 282,696 (37.19) | 249,818 (32.86) |
|  | HbA1c ≥9% | 100,330 (13.20) | 104,628 (13.76) |
|  | Hypoglycemia <70 mg/dL | 57,214 (7.53) | 38,098 (5.01) |
| Model IV | Outcome | COVID-19 group (n=738,546), Events (%) | Control group (n=738,546), Events (%) |
|  | Overall diabetic retinopathy | 10,441 (1.41) | 6,419 (0.87) |
|  | Non-proliferative DR | 6,503 (0.88) | 3,912 (0.53) |
|  | Proliferative DR | 1,386 (0.19) | 855 (0.12) |
|  | Diabetic macular edema | 2,923 (0.40) | 1,626 (0.22) |
|  | Hyperglycemia ≥180 mg/dL | 297,810 (40.32) | 257,505 (34.87) |
|  | HbA1c ≥9% | 108,217 (14.65) | 110,788 (15.00) |
|  | Hypoglycemia <70 mg/dL | 60,313 (8.17) | 38,651 (5.23) |

Data are presented as number of events (%). Model I included only patients who survived during follow-up. Model II included only patients with at least one healthcare visit during follow-up. Model III used a 1-year landmark design. Model IV used a 2-year landmark design. Propensity score matching was performed separately within each sensitivity and landmark analytic cohort using the eligible population for that analysis. DR, diabetic retinopathy; HbA1c, hemoglobin A1c.

**Supplemental Table 6. Sensitivity analyses for the association between COVID-19 and study outcomes in patients with at least one eye-care visit during follow-up.**

| Outcome | COVID-19 group (n=53,425), Events (%) | Control group (n=53,425), Events (%) | HR (95% CI) | Log-rank p value |
| --- | --- | --- | --- | --- |
| Overall diabetic retinopathy | 5,597 (10.48) | 3,903 (7.31) | 1.54 (1.47–1.60) | <0.001 |
| Non-proliferative DR | 4,367 (8.17) | 2,991 (5.60) | 1.55 (1.48–1.63) | <0.001 |
| Proliferative DR | 838 (1.57) | 520 (0.97) | 1.68 (1.50–1.87) | <0.001 |
| Diabetic macular edema | 1,888 (3.53) | 1,184 (2.22) | 1.67 (1.56–1.80) | <0.001 |
| Hyperglycemia ≥180 mg/dL | 30,828 (57.70) | 27,286 (51.07) | 1.27 (1.25–1.29) | <0.001 |
| HbA1c ≥9% | 14,395 (26.94) | 14,378 (26.91) | 1.02 (1.00–1.05) | 0.053 |
| Hypoglycemia <70 mg/dL | 5,460 (10.22) | 3,629 (6.79) | 1.59 (1.53–1.66) | <0.001 |
| Age-related cataract | 25,528 (47.78) | 23,896 (44.73) | 1.13 (1.11–1.15) | <0.001 |
| Any healthcare visit | 53,425 (100.00) | 53,424 (100.00) | 1.18 (1.17–1.20) | <0.001 |
| Mortality | 2,185 (4.09) | 1,412 (2.64) | 1.63 (1.53–1.75) | <0.001 |

Data are presented as number of events (%). This sensitivity analysis was restricted to patients with at least one eye-care visit during follow-up. Hazard ratios were estimated using Cox proportional hazards models. Log-rank p values were derived from Kaplan–Meier analyses. CI, confidence interval; COVID-19, coronavirus disease 2019; DR, diabetic retinopathy; HbA1c, hemoglobin A1c; HR, hazard ratio.
